# Silent Bowels From a Silent Bite: A Rare Case of Paralytic Ileus Complicating Plasmodium falciparum Infection

**DOI:** 10.7759/cureus.34061

**Published:** 2023-01-22

**Authors:** Muhammad Ghallab, Mike Chung, Nicholas Zamora, Heng-Tien Aaron Lee, Natalie Balassiano, Salma Abdelmoteleb, Md Gulam Khan, Hazem Abosheaishaa, Kawser Ahmed

**Affiliations:** 1 Internal Medicine, Icahn School of Medicine at Mount Sinai, New York City Health and Hospitals, New York, USA; 2 Internal Medicine, The New York Institute of Technology College of Osteopathic Medicine, New York, USA; 3 Internal Medicine, St. George's University School of Medicine, New York, USA; 4 Internal Medicine, Icahn School of Medicine at Mount Sinai, Queens Hospital Center, New York, USA; 5 Internal Medicine, Cairo University School of Medicine, Cairo, EGY; 6 Internal Medicine/Gastroenterology, Cairo University, Cairo, EGY; 7 Internal Medicine, Icahn School of Medicine at Mount Sinai, Queens hospital center, New York, USA

**Keywords:** malaria clinical features, gastrointestinal ileus, falciparum malaria, : malaria, paralytic ileus

## Abstract

Malaria is a life-threatening, parasitic disease that continues to infect millions of people, especially in endemic regions. Despite advancements in malaria treatment, treating the disease remains challenging. One major challenge is identifying the disease from its unconventional manifestations. Therefore, recognizing its unusual clinical presentations is imperative in early detection and management with a better prognosis. This case report highlights the unique finding of paralytic ileus from a patient with confirmed malaria. Further investigation on the concurrence between paralytic ileus and malaria may aid in identifying the disease and subsequent improvement in treatment.

## Introduction

Malaria is an ancient disease that has impacted human history. Although we have made great strides in treating malaria, the disease continues to affect millions worldwide. Most malaria cases and deaths come from Africa, and the disproportionate amount of the deaths in the region is accounted for by children under five years of age. Cardinal signs of malaria are often unspecific, commonly presenting with headache, fever, rigors, anemia, splenomegaly, lethargy, nausea, vomiting, and diarrhea [1]. As a result, malaria can be confused with other diseases, making travel history crucial in diagnosis. Extensive research has been conducted to identify symptoms that are more characteristic of malaria. Although not ubiquitous, symptoms such as paroxysms and anemia, along with its other cardinal signs, can aid in identifying malaria. Few malaria cases have shown that the disease may present with unusual symptoms. Some unique cases demonstrated that malaria could manifest in gastrointestinal symptoms, such as intestinal obstruction [1,2]. Such unconventional manifestations may hinder prompt identification and lead to delayed treatment. The absence of adequate therapy promptly can lead to fatal complications. Identifying unique GI presentations of malaria will result in a better prognosis for patients and aid in finding pathognomonic features of malaria. In this report, we discuss the case of a 27-year-old African male who was found to have ileus in the setting of confirmed malaria. We aim to highlight a rare case of ileus in malaria to aid in early diagnosis and management.

## Case presentation

A 27-year-old male with a past medical history of recurrent malaria infection presented to the emergency department with a three-day history of high-grade fevers, chills, and generalized body aches. He also experienced three episodes of non-bloody, non-bilious emesis. He reported no bowel movements since the symptoms began and poor oral intake due to the vomiting. Two weeks before the onset of his symptoms, he returned to the United States after spending one month in Guinea in West Africa. While there, he spent time outdoors and recalls being bitten by mosquitoes on several occasions. He reports having malaria multiple times as a child, with the last infection occurring approximately 12 years ago.

**Figure 1:**
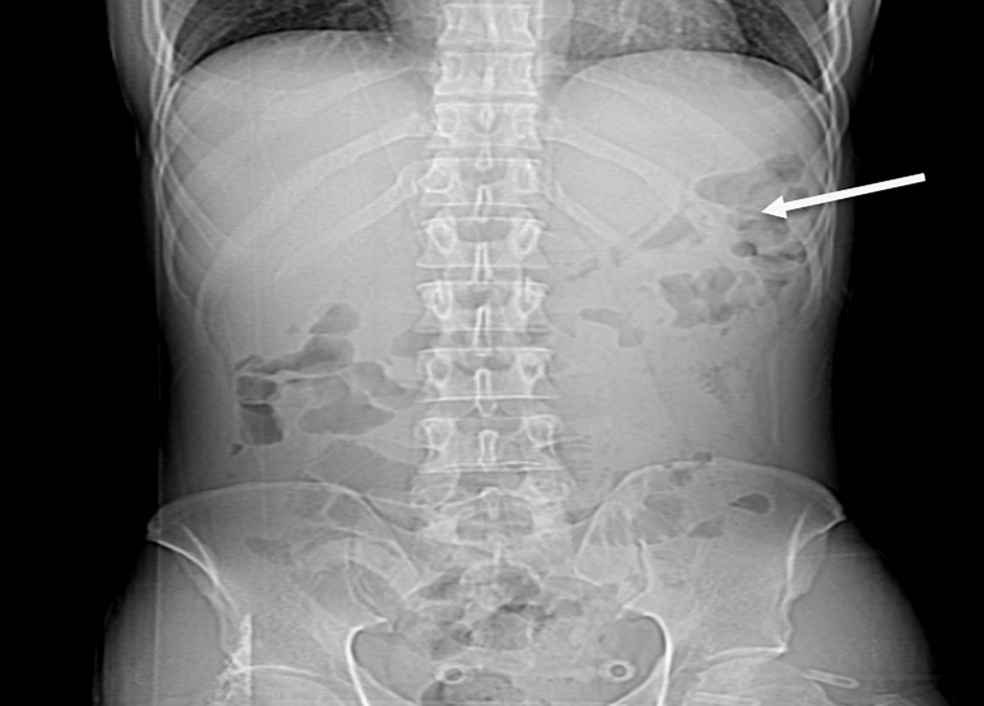
Abdominal X-ray shows scattered air-fluid levels within the small bowel (white arrow).

**Figure 2:**
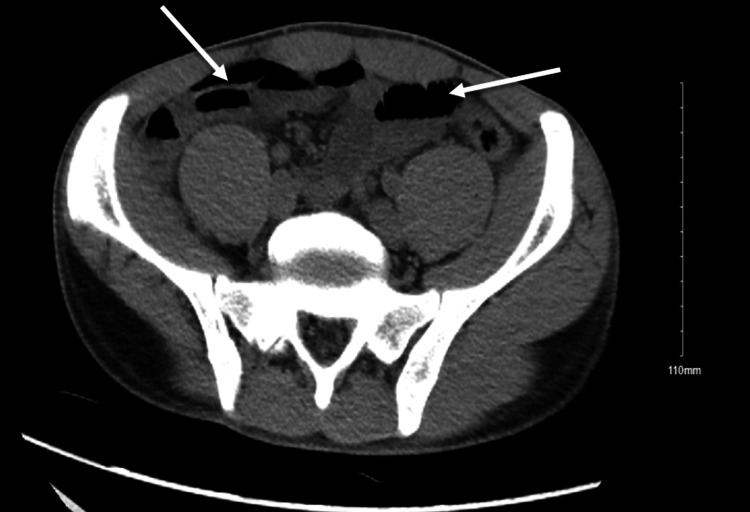
CT abdomen and pelvis without contras (axial section) shows air-fluid levels scattered within the bowel (white arrows).

Upon presentation to the emergency department, the patient was noted to have a fever of 101^o^F (38.3^o^C) and tachycardia (122 bpm). Other vital signs were within normal limits (blood pressure: 102/65 mmHg, respiratory rate: 16 cycles/minute, oxygen saturation on room air: 98%). The physical exam was unremarkable. Initial laboratory findings include mild leukopenia (3.97x103/mcL) with 33% bands and 9% lymphocytes, thrombocytopenia (60x103/mcL), mild transaminitis (aspartate aminotransferase: 62 U/L, alanine aminotransferase: 46 U/L, alkaline phosphatase: 39 U/L, total bilirubin: 1.40 mg/dL), elevated procalcitonin (0.46 ng/mL), elevated lactate (1.9 mmol/L) and negative for SARS-CoV-2, influenza A and B, and respiratory syncytial virus (RSV). The basic metabolic panel was within the normal range (blood urea nitrogen: 15 mmol/L, creatinine 0.9 mg/dl, potassium level: 4.1 mmol/L, and magnesium level: 2.1 mg/dl). Chest X-ray and CT head without contrast did not reveal any abnormalities. X-ray abdomen and CT abdomen/pelvis revealed air-fluid levels within the large and small intestines suggestive of ileus (Figures 1-2). There was no evidence of bowel obstruction. A right upper quadrant ultrasound was done and was within normal apart from mild hepatomegaly. 

While in the emergency department, the patient was treated with intravenous vancomycin and piperacillin-tazobactam. The patient was admitted to the hospital for systemic inflammatory response syndrome (fever, tachycardia, leucopenia) with bandemia and possible sepsis (no obvious source of infection). Peripheral blood smear revealed evidence of malaria rings, and the blood parasites test confirmed Plasmodium falciparum infection. Additionally, his hepatitis B panel revealed a positive hepatitis B surface antigen (HBsAg), total antibody to hepatitis B core antigen (anti-HBc), and anti-HBe, suggestive of chronic disease. While he has no prior hepatitis B diagnosis, he endorsed that multiple family members had been treated for hepatitis B in the past. During the hospital course, the patient became afebrile with the improvement of the bandemia (bands cell decreased to 1%); he started to tolerate the diet gradually, with the improvement of the bowel sounds indicating clinical improvement of the ileus. Per Infectious Disease’s recommendation, he was started on oral artemether-lumefantrine therapy and referred to an outpatient gastroenterologist for chronic hepatitis B management.

## Discussion

Plasmodium falciparum malaria can cause a broad range of multisystemic complications [3-5]. Significant complications include cerebral malaria, severe anemia, disseminated intravascular coagulation, cardiovascular failure, acute kidney injury, and metabolic acidosis. Malaria has been shown to cause gastrointestinal complications such as hepatic dysfunction, splenic rupture, and acute pancreatitis [6]. Recent reports have indicated malaria can also rarely cause intestinal obstruction in both adults and children [2,6]. Although the exact mechanism remains unclear, there have been suggestions that many of these complications may be attributed to common malarial erythrocyte rosetting and Plasmodium falciparum’s unique feature of cytoadherence [5], which allows infected erythrocytes to become “sticky” and bind to the endothelium [3,4]. Furthermore, significantly elevated von Willebrand factor multimers and ADAMTS13 insufficiency associated with severe Plasmodium falciparum malaria may explain early platelet consumption and microangiopathic organ damages [7].

Ileus is rarely if at all, discussed as a complication of malaria. Our unexpected, novel discovery further challenges our understanding of the disease’s underlying pathogenesis for its gastrointestinal complications. While ileus is most commonly associated as a postoperative complication, it can develop from infectious etiologies such as Yersinia enterocolitica and Campylobacter sp. [8]. Furthermore, other parasitic pathogens, such as Strongyloides species, have also been shown to cause paralytic ileus [9].

Understanding how malaria can cause ileus remains inconclusive. A brief review of the paralytic ileus pathogenesis suggests several possibilities [8]. About our patient, the hematogenous spread of malaria may cause inflammation of the gastrointestinal tract, leading to compromised peristalsis. While peritonitis from splenic rupture may cause ileus, our patient did not have an enlarged spleen or splenic rupture.

Furthermore, ileus may arise as a sequela of intestinal obstruction, which has been demonstrated to be another rare complication of malaria. It is also possible that ileus is a unique complication of Plasmodium falciparum due to its ability to cause cytoadherence and erythrocyte rosetting [5], leading to bowel ischemia. As previously explored, bowel disorders associated with Plasmodium falciparum infection have been linked to coagulation cascade activation from erythrocyte membrane changes [1,7]. Conversely, a translational study demonstrated that Plasmodium berghei causes damage to the intestinal epithelium and vascular occlusions, leading to villi destruction in infected mice [10]. Although not definitive to Plasmodium falciparum infection, the findings suggest Plasmodium species can result in bowel complications due to bowel wall dysfunction and ischemia.

## Conclusions

Since malaria has been demonstrated to cause gastrointestinal complications such as hepatic dysfunction, splenic rupture, and acute pancreatitis, an abdominal CT is crucial in identifying any complications caused by malaria, especially when patients present with signs of gastrointestinal problems; in this case, it was emesis and constipation. In rare instances, functional bowel obstruction and paralytic ileus can be found. As cases of paralytic ileus are primarily self-limiting, cases of paralytic ileus due to malaria should resolve with treatment and resolution of malaria, as was seen in our patient and previously reported patients.
